# Traditional Chinese medicine syndrome differentiation and treatment by stages of Parkinson’s disease: study protocol for a multicentre, randomized, double-blind, placebo-controlled clinical trial

**DOI:** 10.1186/s13020-022-00625-4

**Published:** 2022-06-13

**Authors:** Yuqing Hu, Sichun Gu, Xiaolei Yuan, Hui Li, Canxing Yuan, Qing Ye

**Affiliations:** Encephalopathy Department, Longhua Hospital Affiliated to Shanghai University of Traditional Chinese Medicine, No. 725, South Wanping Road, Shanghai, China

**Keywords:** Parkinson’s disease, Traditional Chinese medicine, Syndrome differentiation

## Abstract

**Background:**

Parkinson’s disease (PD) is a progressive neurodegenerative disease common in aged populations. Classified by Hoehn & Yahr stages, patients are often divided into mild/early stage, moderate/middle stage, and advanced/late stage. With disease progression, PD shows high heterogeneity in each stage. Based on traditional Chinese medicine (TCM) syndrome differentiation theory and our previous works, we found that during the early stage, the main syndrome is Yin deficiency of the liver and kidney; during the moderate stage, the main syndromes are phlegm heat and wind stirring and blood stasis and wind stirring; and during the late stage, the dominant syndromes are deficiency of Yin and Yang and deficiency of Qi and blood. Hence, we proposed a new model of TCM treatment by the stage of PD. Based on Shudi Pingchan formula, an experimental formula of our team, we developed Ziyin Pingchan formula, Jiedu Pingchan formula, and Fuzheng Pingchan formula to treat each stage. This study is designed to evaluate the therapeutic effect of treating Parkinson’s disease by stages using traditional Chinese medicine and to provide an evidence base for forming a standardized scheme of diagnosis and treatment.

**Methods:**

This study is designed as a multicentre, randomized, double-blind, placebo-controlled clinical trial. Patients will be stratified into 3 subgroups according to Hoehn & Yahr stage; 172, 168, and 72 participants will be required to be in the mild PD, moderate PD, and advanced PD subgroups, respectively, and will be randomized into the treatment or control group at a 1:1 ratio. The mild PD subgroup will receive a 48-week intervention, and the other 2 groups will receive a 24-week intervention. All groups will have a follow-up visit 12 weeks after starting the intervention. The intervention group will receive the Ziyin Pingchan formula, Jiedu Pingchan formula, or Fuzheng Pingchan formula, and the control group will receive the corresponding placebo. The primary outcomes will be the first addition of levodopa for the mild PD subgroup, the duration of the “OFF” period for the moderate PD subgroup, and the Parkinson's Disease Questionnaire (PDQ-39) for the advanced PD subgroup. The secondary outcomes will also be verified by subgroups, including the Unified Parkinson’s Disease Rating Scale (UPDRS), Parkinson’s Disease Sleep Scale-2 (PDSS-2), scales for Outcomes in Parkinson’s Disease—Autonomic (SCOPA-AUT), and the nonmotor symptom scale (NMSS).

**Expected outcomes:**

To our knowledge, this is the first trial to combine TCM syndrome differentiation with PD clinical stages and put it into clinical practice. The results of this trial will provide clinical evidence for the therapeutic effect of TCM formulas on PD patients of all stages and help build a new TCM treatment by stage model of PD.

*Trial registration*: This trial is registered at the Chinese Clinical Trial Registry (http://www.chictr.org.cn/). Registration number: ChiCTR2200056373, Date: 2022–02-04, version 1.

## Background and rationale

Parkinson’s disease (PD) is a highly sporadic neurodegenerative disease common in the aged population [1]. With an annual incidence ranging from 10 to more than 20 per 100,000 individuals [2], the worldwide disease burden could exceed 17 million by 2040 [3], and the Chinese Parkinson population could be 5 million by 2030. As a progressive disease leading to a high rate of disability, PD imposes a great burden on society and caregivers, which is becoming substantially more severe due to the ageing of society [4].

To build a better picture of disease progression, the Hoehn & Yahr stage [5] has been created and is widely used in clinical practice, and studies have shown that the Hoehn & Yahr stage is correlated with both motor symptoms (MSs) and nonmotor symptoms (NMSs) [6]. To facilitate clinical studies, PD patients are often classified into different stages according to the Hoehn & Yahr stage, namely staged ≤ 2 as mild/early, 2.5 and 3 as moderate/middle, and > 3 as advanced/late [7]. Treatment plans are also made based on these stages after comprehensive considerations.

Since the existence of MS and NMS occurs throughout the disease duration, current guidelines recommend long-term management therapy, emphasizing the distinct particularities of different stages of the disease. The key feature of the mild stage is considerable progression; during this stage, neuroinflammation and pyroptosis can be found, which promotes the loss of dopaminergic neurons [8]. With disease progression and an increased dose of levodopa, patients are prone to suffer from motor fluctuations and dyskinesia, mainly described as a wearing-off period and peak-dose dyskinesia. As the disease advances, α-synuclein pathology spreads to higher centres in the brain, involving the limbic system, amygdala, neocortex, and prefrontal lobe [9]. With the participation of the side effects of medication and peripheral disorders, these pathogenesis eventually lead to a grievous “OFF” time, disabling motor complications and a wide range of NMSs, including cognitive impairment, sleep disorders, and autonomic nerve dysfunction. Hence, clinicians always eventually shift their focus to dealing with quality of life (QoL).

Pharmacological therapy is always a challenge at all stages of PD. First, corroborating the necessity of early initiation of disease-modification therapy, the present problem is the absence of disease-modifying drugs [10]. Monoamine oxidase-B inhibitors (MOA-BI), including selegiline and rasagiline, have been reported to improve the dopamine-deficient state and the activity of some antioxidant, antiapoptotic, and neurotrophic factors, but a clinical neuroprotective effect lacks high-level clinical evidence [11]. Second, levodopa, the predominant anti-Parkinson drug, is now 65 years old [12]. Studies have indicated that levodopa-induced motor fluctuation occurs in more than half of long-term medicated PD patients [13]. In addition to dopaminergic neuron loss, pulsatile delivery of high-dose levodopa causes plasma concentration fluctuation following phasic stimulation of dopamine receptors, further worsening the function of dopaminergic receptors in the postsynaptic membrane [14]. Although dopamine agonists (DAs), such as pramipexole, ropinirole, apomorphine and rotigotine, can increase the “ON” time and reduce the “OFF” time [15, 16], they cause dopaminergic side effects, including orthostatic hypotension, dizziness, nausea, somnolence, and impulse control disorders [10]. Last, in the advanced stage, severe neuron loss forces patients to take high-dose, combined pharmacological therapy and even undergo invasive treatments to maintain a relatively stable situation [17]. Otherwise, levodopa-induced dyskinesia and the combinations of anti-Parkinson medicines causes conflicts between effective dosages and clinical responses during the treatment of moderate to advanced PD patients, making treatment more complex. Due to this complicated situation, an increasing number of patients are starting to receive traditional Chinese medicine (TCM) treatment, and studies have indicated that TCM therapy can improve both motor and nonmotor symptoms in PD patients [18].

Our team has been working on TCM treatment of PD for decades. In past clinical practice, we attributed the basic mechanism of Parkinson's disease to "liver wind", and the pathogenesis is root vacuity and tip repletion. Deficiency of Zang-fu organ function obstructs the formation and circulation of Qi and blood and causes blood stasis and phlegm, whose long-term accumulation transforms to toxin. Past analyses indicated that the most common syndromes of PD are Yin deficiency of liver and kidney (YDLK), deficiency of Qi and blood (DOQB), phlegm heat and wind stirring (PHWS), blood stasis and wind stirring (BSWS), and deficiency of Yin and Yang (DOYY) [19].

**Fig. 1:**
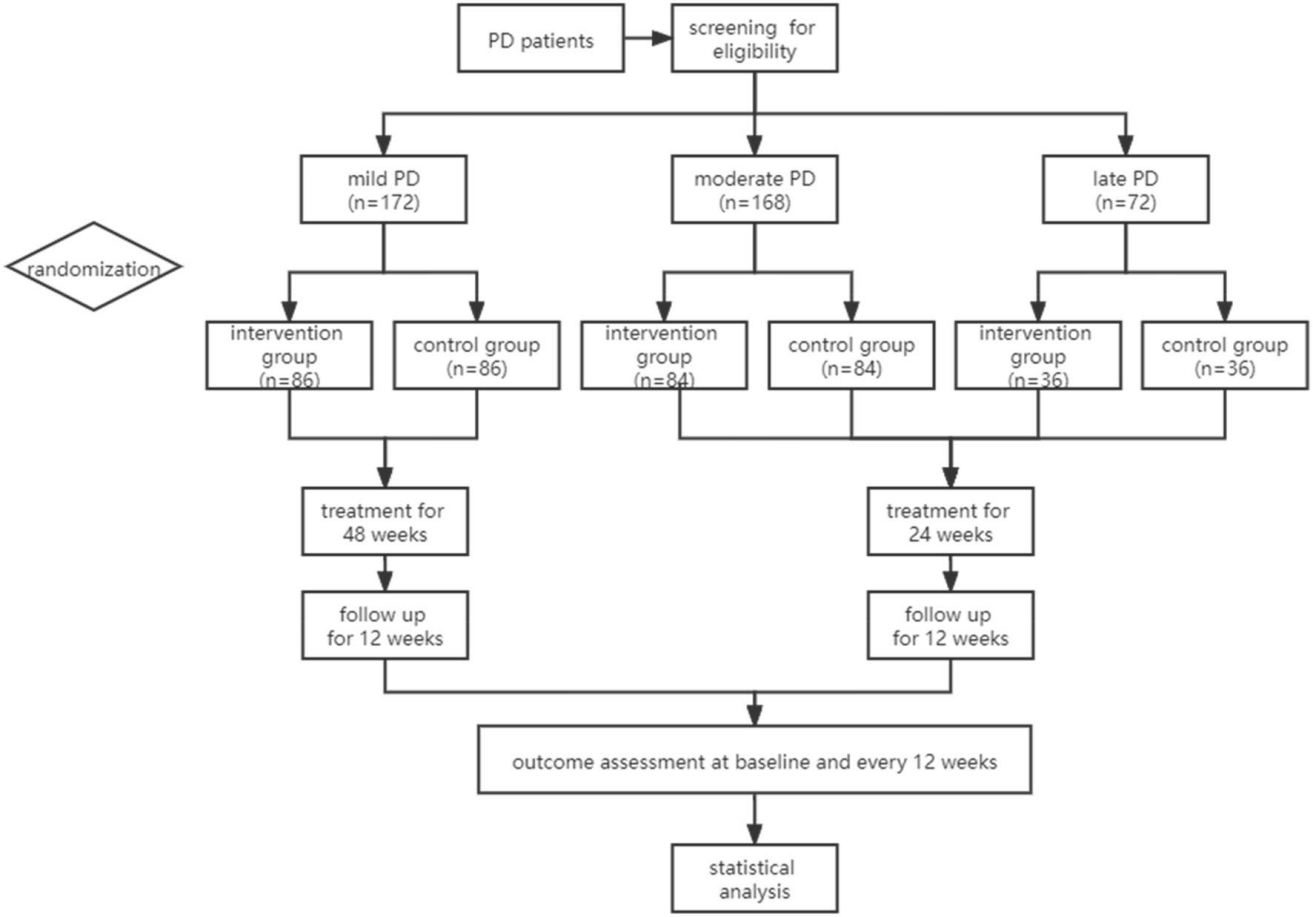
TCM pathogenesis of PD

Our unpublished works further correlated the PD clinical stages to TCM syndromes and found that there are relatively uniform syndromes during each stage of disease development. During the early stage, the main syndrome is YDLK, and the pathogenesis is Yin deficiency of the liver and kidney. Kidney water fails to nourish liver wood; thus, liver wind stirs internally, so symptoms occur, “wind being mobile and changeable” [20]. This stage experiences relatively high-speed progression. During the moderate stage, the main syndromes are PHWS and BSWS, and long-term accumulation of phlegm and blood stasis, together with medical toxin, are the main source of toxin damage to the brain, which encumbers treatment. During the late stage, the dominant syndromes are DOYY and DOOB. Because of the mutual rooting of Yin and Yang, Yin impairment involves Yang, and toxin simultaneously damages vital Qi, finally causing a deficiency of both Yin and Yang. A schematic diagram of the pathogenesis is shown in Fig. 1.

Following the therapeutic methodology of nourishing the liver and kidney, dredging collaterals, and detoxification, our team established the Pingchan formula in the past decade. The Pingchan formula is made up of *Rehmanniae radix* praeparata (Shudihuang) 15 g, *Lycii fructus* (Gouqizi) 12 g, *Taxilli herba* (Sangjisheng) 15 g, *Gastrodiae rhizoma* (Tianma) 9 g, *Paeoniae radix* alba (Baishao) 15 g, *Bombyx batryiticatus* (Jiangcan) 9 g, Scorpio (Quanxie) 3 g, and scolopendra (Wugong) 3 g. Our past studies have proven its efficacy in reducing the occurrence of motor complications and mitigating dyskinesia and improving depression in mild and moderate PD patients [21, 22]. Additionally, our previous study indicated that Pingchan granules can alleviate inflammation and apoptosis in nigral cells and dopaminergic neurons in PD mice induced by 1-methyl-4-phenyl-1,2,3,6-tetrahydropyridine (MPTP) by regulating the c-Jun N-terminal protein kinase (JNK) pathway in the substantia nigra [23]. In addition, considering the side effects of long-term use of levodopa, Pingchan granules inhibit the hyperphosphorylation of extracellular signal-regulated kinase and downregulate the expression of anti-apoptotic genes worsened by levodopa [24], simultaneously upregulating dopamine D2 receptor gene expression, whose overinhibition has been proven to be the main mechanism of levodopa-induced dyskinesia [25].

On the basis of the Pingchan formula and our syndrome differentiation by stage theory, we proposed a new model of TCM treatment by stage of PD. Based on the general principle of nourishing liver Yin and kidney Yin, we introduced detoxification and supporting vital Qi, respectively, to the treatment of moderate and advanced stages, aiming at delaying disease progression during the early stage, dealing with complications during the moderate stage, and improving the QoL during the late stage. We removed scolopendra from the Pingchan formula to produce the Ziyin Pingchan formula for the mild stage to nourish the liver Yin and kidney Yin and to slow the process of accumulation of toxin in the brain. Ziyin Pingchan formula was used as the foundation, to which we added *Arisaematis rhizoma* preparatum (Zhitiannanxing) 15 g and *Curcumae rhizoma* (Ezhu) 9 g to reduce drug toxin, noxious blood stasis, and phlegm toxin, namely, the Jiedu Pingchan formula for the moderate stage, and *Astragali radix* (Huangqi) 30 g, *Cistanches herba* (Roucongrong) 30 g to nourish the kidney Yang and vital Qi to additionally moisten the intestines and urge purgation, namely, the Fuzheng Pingchan formula for the late stage. Based on the rationales above, we hypothesize that every formula will succeed in treating each stage of PD.

Our treatment by stages therapy is a long-term management treatment that covers the key points of every stage of PD based on TCM pathogenesis. In the mild stage, we delay the progression of the disease. In the moderate stage, we improve the symptoms, increase the curative effect and reduce the side effects of anti-Parkinson medicine. In the advanced stage, we improve the QoL. This TCM whole-process management and treatment can delay disease progression, improve the complications caused by Western medicine, and improve the QoL of patients, which is innovative and quite practical.

## Objective

The major objective of this study is to evaluate the therapeutic effect of treating Parkinson’s disease by stages using integrated traditional Chinese and Western medicine methods and to provide an evidence base for forming a standardized scheme of diagnosis and treatment. In particular, this study aims to (1) determine the therapeutic effect of the Ziyin Pingchan formula for postponing progression and improving the symptoms of mild PD; (2) clarifying the efficacy of the Jiedu Pingchan formula for improving clinical symptoms and preventing and treating the motor complications of moderate PD; and (3) elucidating the effect of the Fuzheng Pingchan formulaformula in improving QoL in advanced PD.

## Methods/design

### Study setting

**Fig. 2:**
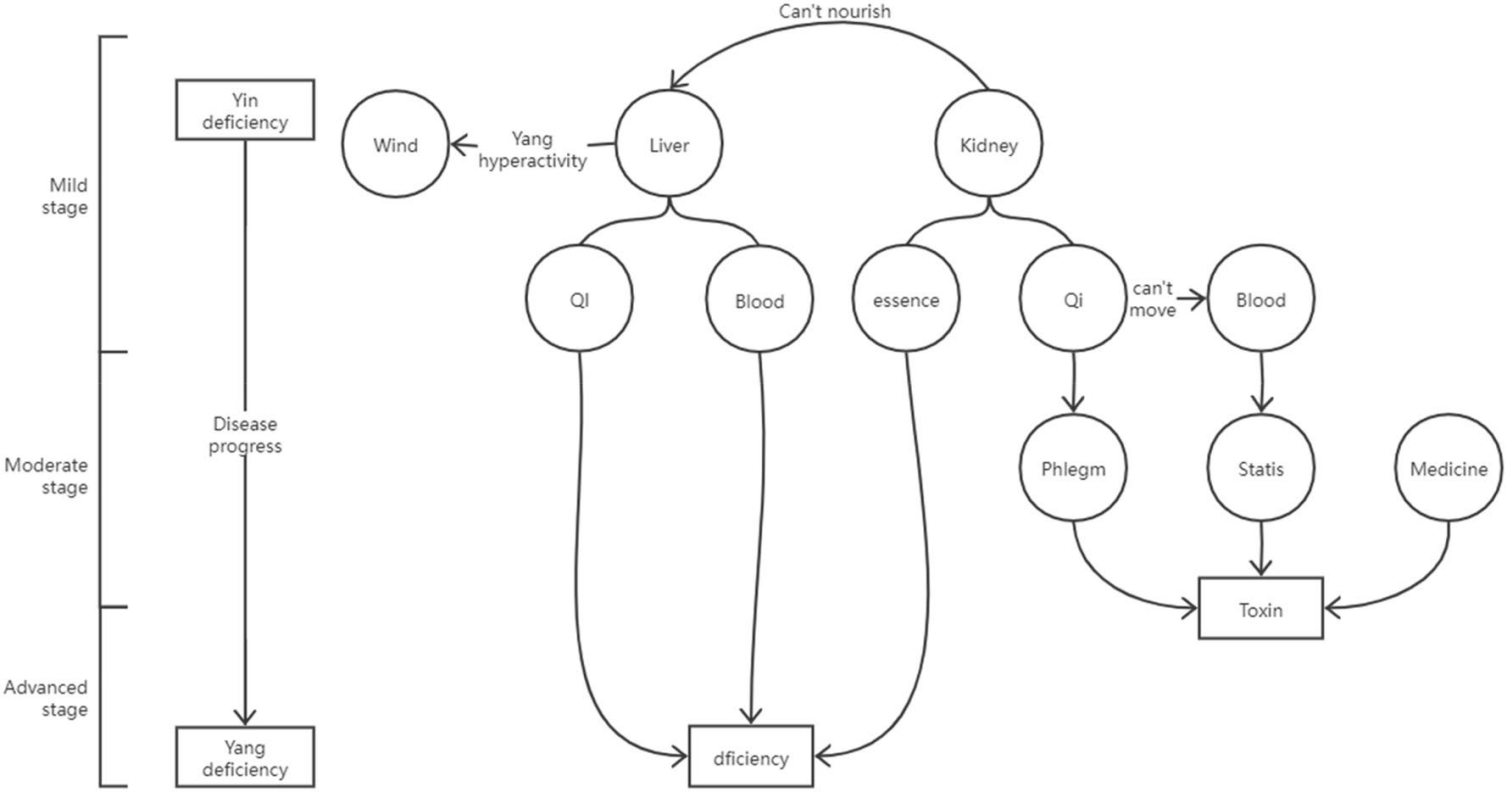
Flowchart of the study

This study is a multicentre, randomized, double-blind, placebo-controlled clinical trial. The study will be simultaneously carried out in seven centres in China, including Longhua Hospital Affiliated to Shanghai University of Traditional Chinese Medicine, Xinhua Hospital Affiliated to Shanghai Jiao Tong University Medical College, Shuguang Hospital Affiliated to Shanghai University of Traditional Chinese Medicine, No 1 People’s Hospital Affiliated to Shanghai Jiao Tong University Medical College, Yueyang Hospital of Integrated Traditional Chinese and Western Medicine affiliated to Shanghai University of Traditional Chinese Medicine, Ruijin Hospital Affiliated to Shanghai Jiao Tong University Medical College, and Yangzhi Rehabilitation Hospital Affiliated to Tongji University. This study is divided into 3 substudies. PD patients diagnosed at different stages will be screened into different units: mild PD unit, moderate PD unit, and advanced PD unit. The mild PD unit will receive a 48-week therapy and 12-week follow-up, and face-to-face assessments will be conducted at baseline (week 0), week 12, week 24, week 36, week 48, and week 60. The other two units will receive a 24-week therapy and 12-week follow-up, and face-to-face assessments will be conducted at baseline (week 0), week 12, week 24, and week 36. Recruitment for the study will be launched in April 2022 and it is anticipated to finish before July 2024. The flow chart of the trial is shown in Fig. 2.

This protocol is written following the Standard Protocol Items for Clinical Trials with Traditional Chinese Medicine 2018 (SPIRIT-TCM Extension 2018). The study was approved by the Research Ethics Committee of the Longhua Hospital Affiliated with the Shanghai University of Traditional Chinese Medicine (2022LCSY002) and will be conducted following the Declaration of Helsinki, and the final study results will be reported following the Consolidated Standards of Reporting Trials (CONSORT).

### Randomization

Stratified group randomization will be applied in this study. Allocation will be applied by block randomization stratified by study centre with block sizes of 4 using SPSS 25.0 software, and the subjects will be randomly divided into the intervention group or control group (Seed Number: 202106151) in a 1:1 ratio. The randomization sequence will be kept in sealed opaque envelopes and will be prepared and preserved by the pharmaceutical company. Researchers in charge of enrolment and evaluation will be blinded to the patient allocation. After the participants are enrolled, they will be given an enrolment number by the sequence they take part in the study, and then they will be given the investigational medicinal product (IMP). An emergency unblinding envelope will be set for each blind code, indicating to which group the subject is assigned and what medication intervention the participant is receiving. Those sealed opaque envelopes will be sent to each study centre together with the study medications of the corresponding blinding code. Each centre will assign a research assistant responsible for allocating the drugs to the participants, and emergency unblinding envelopes will be kept by them.

### Blinding

Blinding codes of granule and placebo will be prepared according to the results of randomization, and the pharmaceutical company is responsible for labelling the packages of IMPs. When all data collection is finished, the research data will be input into an established electronic database and will undergo blinded review by statistical professionals and will be locked if affirmed accurately. Then, primary unblinding will be performed to establish Group A and Group B in each unit. After the completion of the final statistical analysis, second-level unblinding will be performed to reveal the intervention group and control group. Both unblindings will be executed by the responsible investigator. The blinding codes will be kept strictly unrevealed during the research.

### Recruitment

Participants will be recruited among PD patients who visit both the clinic and hospital of the Department of Neurology, and announcements will be distributed through social media, posters in hospitals and university websites. Patients who are interested in enrolling in the study can contact the investigators through telephone, WeChat, e-mail, or at the clinic. After preliminary screening and explanation of the study, potential candidates will be invited to further face-to-face screening by trained neurological physicians and Chinese medicine practitioners. Eligible participants will sign the Informed Consent Form (ICF) and enter the trial.

### Eligibility criteria

Participants must fulfil the diagnostic criteria of clinically probable PD according to the 2015 Movement Disorder Society (MDS) criteria. Patients will be screened according to the inclusion and exclusion criteria listed in Table 1. Patients of all stages are allowed to take anti-Parkinson treatment given by neurological physicians according to guidelines. The treatment should be at a relatively stable dosage for at least 3 months prior to the intervention, and their clinical features should also be relatively stable; thus, no plan should be made to change their existing treatment during follow-up.

**Table 1:** Eligibility criteria

Inclusion criteria	
Age between 30 and 80, regardless of gender	
For participants who are receiving anti-Parkinson treatment (levodopa preparations, dopamine agonists, monoamine oxidase inhibitors, catechins-o-methyl transferase (COMT) inhibitor, anticholinergic drugs, etc.), the dosage of the anti-Parkinson drug is requested to be relatively stable for at least 3 months before this clinical trial, and no plan has been made to change the existing treatment in the next 3 months	
Voluntarily participate and sign informed consent	
Exclusion criteria	
Patients with Parkinson Syndrome (PS) or Parkinsonism-Plus syndrome (PPS)	
Pregnant or lactating women, history of drug or alcohol abuse	
Severe cognitive impairment (Montreal Cognitive Assessment scale (MoCA) < 20 points), active depression or psychosis and/or under antidepressant or antipsychotic medication, severe sequelae of stroke and other systemic diseases affecting the heart, lung, liver or kidney	
Participating in other clinical studies or have participated in other clinical study within the previous 30 days	

Grouping will be conducted according to the Hoehn & Yahr stage at baseline. Stage ≤ 2 will be classified into the mild PD group, stage 2.5 and 3 into the moderate group, and stage ≥ 4 into the advanced group [7]. For patients in the moderate group, at least one of the following motor complications should be reported: wearing-off phenomenon, peak-dose dyskinesia, biphasic dyskinesia, dysmyotonia, and on–off phenomenon. No extra TCM syndrome differentiation will be applied since the correspondence between the TCM syndrome and clinical stage has been described above.

Participants can withdraw from the study at any time if they perceive poor efficacy or adverse reactions. Subjects with poor compliance, serious comorbidities or complications, or serious adverse events will be persuaded to quit the study. These subjects will be treated as drop-out cases, and the time and reason for drop-out should be stated. Once the subject drops out, investigators should try to contact the subject as much as possible to inquire about the reason and complete the assessments. The timing of the last medication should also be recorded.

### Intervention

The three formulas are alternatives for the intervention differentiation among the groups. Ziyin Pingchan Decoction includes seven herbs and worms, namely, *Rehmanniae radix* praeparata (Shudihuang) 15 g, *Lycii fructus* (Gouqizi) 12 g, *Taxilli herba* (Sangjisheng) 15 g, *Gastrodiae rhizoma* (Tianma) 9 g, *Paeoniae radix* alba (Baishao) 15 g, *Bombyx batryiticatus* (Jiangcan) 9 g, and Scorpio (Quanxie) 3 g. Jiedu Pingchan Decoction is the Ziyin Pingchan Decoction with two additional herbs, namely, *Arisaematis rhizoma* preparatum (Zhitiannanxing) 15 g and *Curcumae rhizoma* (Ezhu) 9 g. Fuzheng Pingchan Decoction consists of Ziyin Pingchan Decoction and two extra herbs, namely, *Astragali radix* (Huangqi) 30 g and *Cistanches herba* (Roucongrong) 30 g. The doses of each herb and worm were determined according to The Chinese Pharmacopoeia (Ch. P) 2020 edition and fixed by clinical experience within a safe dose [26, 27].

The placebo will be made from maltodextrin and a 1/10 concentration of the corresponding decoction, which has no therapeutic effect [28]. The form, colour, smell, and solubility of the placebo will simulate the intervention medicine. The IMPs will be processed into granules by water extraction, separation, concentration, drying and granulation by Sichuan Neo-Green Pharmaceutical Technology Development Co., Ltd., and 8 g will be packed per sachet. The production and processing of the decoctions will be conducted in accordance with the Ch. P, and the processing and filling of the granules will be carried out in standardized clean production workshops. The dosage regimen is one package of granules in warm water twice a day, 30 min after breakfast and dinner. Every 56 sachets will be packed in a transparent package with a randomization code label for one month of treatment. Participants will be given the next 3 packages of IMPs at every follow-up visit. The last of the interventions will be 48 weeks for the mild PD unit and 24 weeks for the other 2 units. Participants will be asked to bring all leftover drugs to every follow-up visit to check their compliance.

If a serious adverse event or allergic reaction happens and is of concern relative to the research medication, the emergency envelope will be opened by the principal investigator of the centre. Once opened, research on that subject will be discontinued. Then, the time and reason for the emergency unblinding will be recorded in detail in the case report form (CRF). The patient will receive corresponding standardized treatment. Simultaneously, the relevant departments, including the ethics committees of each study centre, should be informed within 24 h.

### Therapy combination

Participants who had not previously taken anti-Parkinson medicine (levodopa preparations, dopamine agonists, monoamine oxidase inhibitors, catechins-o-methyl transferase (COMT) inhibitor, anticholinergic drugs, etc.) should postpone the application of Western medicine and take IMPs. Patients who have already received anti-Parkinson treatment should continue to take them on the basis of the original dosage. TCM and proprietary Chinese medicine, except for the prescribed IMPs, are prohibited during the observation period. Reduction of the dosage of anti-Parkinson medication is allowed due to the variations of the disease condition. However, if the symptoms do not improve significantly and they affect the participant’s daily life and the Unified Parkinson's Disease Rating Scale (UPDRS) total score is 3 points higher than at baseline, the only anti-Parkinson medication that can be added is levodopa and benserazide hydrochloride tablets (Madopar, 0.25 g*40 s, Shanghai Roche Pharmaceutical Co., Ltd.); other drugs are prohibited, and the time of adjustment and the current dose should be recorded in detail in the CRF.

Necessary medications for comorbidities must be recorded in the CRF, including the generic name, dosage, and regimen of the drug. Participants will be required to bring all medications they are taking to every follow-up visit to check their concomitant medication.

### Outcomes

Our objectives alternate according to the different stages of PD, so we set different outcomes for each unit for the data analyses. A detailed schedule and list of parameters are shown in Table 2.

**Table 2:** Schedule of assessments

Assessments	Group			Study period							O
				Enrolment	Allocation	Intervention				Follow-up	
Timepoint	Mi	Mo	A	−T1	T0	T1	T2	T3	T4	T5	
Enrolment				×							
Eligibility screen	×	×	×	×							
Informed consent	×	×	×	×							
Allocation	×	×	×		×						
Randomization	×	×	×		×						
Intervention											
Ziyin Pingchan formula	×					×	×	×	×		
Jiedu Pingchan formula		×				×	×				
Fuzheng Pingchan formula			×			×	×				
Time of first addition of levodopa	×			×		×	×	×	×	×	P^Mi^
UPDRS	×	×	×	×		×	×	×	×	×	
PDSS-2	×	×		×		×	×	Mi	Mi	×	
ESS	×	×		×		×	×	Mi	Mi	×	
RBDSQ	×	×		×		×	×	Mi	Mi	×	
HAMD	×	×		×		×	×	Mi	Mi	×	
HAMA	×	×		×		×	×	Mi	Mi	×	
SCOPA-AUT	×	×		×		×	×	Mi	Mi	×	
NMSS	×	×	×	×		×	×	Mi	Mi	×	
PDQ-39	×	×	×	×		×	×	Mi	Mi	×	P^A^
Elderly quiver of TCM syndrome classification dysfunction score	×	×	×	×		×	×	Mi	Mi	×	
Duration of “OFF” period		×		×		×	×			×	P^Mo^
Hoehn-Yahr stage		×		×		×	×			×	
WCSS		×		×		×	×			×	
WOQ-9		×		×		×	×			×	
UDysRS		×		×		×	×			×	
AIMS		×		×		×	×			×	
PDYS-26		×		×		×	×			×	
FOGQ		×		×		×	×			×	
CBI			×	×		×	×			×	
MMSE			×	×		×	×			×	
MoCA			×	×		×	×			×	
LED		×		×		×	×			×	
Infection			×	×		×	×			×	
Hospitalization			×	×		×	×			×	
Survival			×	×		×	×			×	
UA	×	×		×		×	×	Mi	Mi	×	
Hcy	×	×		×		×	×	Mi	Mi	×	

*Mi* mild, *Mo* moderate, *A* advanced, *T0* baseline, *T1* week 12, *T2* week 24, *T3* week 36, *T4* week 48, *T * week60, *O* outcome

#### Mild PD

We will use the time of the first addition of levodopa as the primary outcome for the mild PD unit to reveal the progression of the disease. When participants have a 3-point-higher UPDRS III score and have more serious symptoms, their physicians could give them levodopa or increase the dose of levodopa they were taking. The timing will be recorded at every follow-up visit or reported by the participants at any time.

Secondary outcomes will be UPDRS I–IV and total score for disease evaluation, Parkinson’s disease Sleep Scale-2 (PDSS-2), the Epworth Sleeping Scale (ESS), REM sleep behaviour disorder questionnaire (RBDSQ), Hamilton Depression Scale (HAMD), Hamilton Anxiety Scale (HAMA), scales for Outcomes in Parkinson’s disease—Autonomic (SCOPA-AUT), nonmotor symptom scale (NMSS) for assessing NMSs, Parkinson's Disease Questionnaire (PDQ-39) for evaluating QoL, and elderly quiver of TCM syndrome classification dysfunction score for TCM syndrome evaluation, uric acid (UA) and homocysteine (Hcy) for disease prognosis.

#### Moderate PD

For the moderate PD unit, the primary outcome is the duration of the “OFF” period. The curative effect will be defined as “remission” by a ≥ 75% reduction rate, “response” for between 50 and 75%, “partial response” for between 30 and 50%, and “invalid” for below 30%.

Secondary outcomes will be Hoehn Yahr stage, Webster Clinical Symptom Scale (WCSS), UPDRSI-II for disease evaluation, Freezing of Gait Questionnaire (FOGQ) and abnormal involuntary movement scale (AIMS) to estimate MS; Wearing-off-9 Questionnaire (WOQ-9), Unified Dyskinesia Rating Scale (UDysRS), and Parkinson disease dyskinesia scale (PDYS-26) to estimate motor complications; PDSS-2, ESS, RBDSQ, NMSS, HAMD, HAMA, and SCOPA-AUT for assessing NMSs; the PDQ-39 for evaluating QoL; and the elderly quiver of TCM syndrome classification dysfunction score for TCM syndrome evaluation. UA and Hcy for disease prognosis, levodopa equivalent dose (LED), and the percentage of patients who increased their levodopa dosage will be considered in the overall response.

### Advanced PD

We used the PDQ-39 as the primary outcome of advanced PD units to evaluate the QoL of advanced PD patients. The curative effect will be defined as “remission” by a ≥ 75% reduction rate, “response” for between 50 and 75%, “partial response” for between 30 and 50%, and “invalid” for below 30%. Secondary outcomes are Caregivers Burden Inventory (CBI), bed rest, infection, hospitalization, and survival to evaluate QoL obliquely; Hoehn Yahr stage and UPDRS for disease evaluation; NMSS, mini-mental state examination (MMSE), and MoCA for assessing cognitive function; elderly quiver of TCM syndrome classification dysfunction score for TCM syndrome classification; and UA and Hcy for disease prognosis.

### Safety outcomes

Safety outcomes will be assessed at baseline and at the last visit, including red blood cell count (RBC), white blood cell count (WBC), platelet count (PLT), glutamic oxaloacetic transaminase (GOT), glutamic pyruvic transaminase (GPT), serum creatinine (Scr) and electrocardiogram (EKG). Laboratory results will be judged as abnormal if 20% out of the normal range or with supportive signs or symptoms. The WBC and EKG results should be judged based on the patient's specific situation.

#### Sociodemographic factors

Sociodemographic and clinical data, including sex, age, occupation, marital status, educational background, disease duration, and onset age, will be collected by baseline assessment.

### Biological specimen collections

Blood samples will be collected and measured using standard laboratory procedures; all samples will be destroyed after the data are reported.

### Sample size

The minimum necessary sample size by normal approximation was determined by the following formula [29]:

$$n_{1} = n_{2} = 2 \times \left[ {\frac{{\left( {u_{\alpha } + u_{\beta } } \right)\sigma }}{\delta }} \right]^{2} + \frac{1}{4}u_{\alpha }^{2}$$

On the basis of our previous study, the estimated difference in the time of first addition of levodopa between the two groups among mild PD patients was 3.6 months, standard deviation (S.D.) 6.6. The estimated difference in the duration of the “OFF” period between the two groups among moderate PD patients was 4.6 min, S.D. 8.3. The estimated difference in the PDQ-39 score between the two groups among advanced PD patients was 7.3 points, S.D. 8.5. Considering a 5% significance level and a determined 0.10 β value, Uα/2 = 1.96, Uβ = 1.28, we calculated 72 cases are required in each group in the mild PD unit, 84 in the moderate PD unit, and 36 in the advanced PD unit. Considering a 20% drop-out rate, a sample of 172 subjects in the mild PD unit, 168 in the moderate PD unit, and 72 in the advanced PD unit is required, with a total of 412 participants enrolled in the study.

### Statistical methods

Statistical analysis will be performed using SPSS 25.0 software. Measurement data will be presented as the mean ± S.D. Comparisons between two groups will be conducted using Student’s t-test or the Mann–Whitney U test. Quantitative data will be reported as percentages and proportions, and comparisons between two groups will be performed by χ2 or Fisher’s exact tests. The Mann–Whitney U test will be used to compare the ordinal data. Multivariate analysis will be performed using analysis of variance (ANOVA) or generalized estimation equations (GEE). All analyses will use two-tailed tests with a P value of 0.05 as the threshold for significance.

### Data management

Research methods, including CRF, ICF, and reports, will be collected from all collaborating centres after the trial, all documents will be kept in locked safes, and electronic data will have a password to ensure confidentiality. Data monitoring will be the responsibility of the Stas Central of Shanghai University of Traditional Chinese Medicine, the Research Ethics Committee of Longhua Hospital Affiliated with the Shanghai University of Traditional Chinese Medicine. The cooperating centres will be responsible for steering this trial, and endpoint adjudication will be conducted by the Science and Technology Commission of Shanghai Municipality.

### Termination criteria

The trial will be terminated if any of the following happens: (1) the IMP has serious safety problems; (2) It shows that the efficacy of the drug has poor efficiency; (3) important deviations in design or implementation of study protocol makes it difficult to evaluate therapeutic effects; (4) premature termination due to reasons of the sponsor; and (5) the trial is asked to stop by the administration.

## Discussion

PD is a neurodegenerative disease with complex motor and nonmotor symptoms. It has different features at different stages. The mild stage results in circumscribed MS and NMS. In the moderate stage, motor complications and side effects of complicated anti-Parkinson medications occur. In the advanced stage, dyskinesia together with cognitive impairment restricts the patients’ activity and aggravates their burden on caregivers. Hence, the emphasis of treatment should be differentiated by stages.

Current guidelines recommend disease modification therapy for mild PD but lack a phased treatment program for moderate and advanced PD. Simultaneously, combination drug therapy generates drug resistance and side effects, which are exciting to moderate and advanced PD patients. Although synergy between TCM and WM treatments has been proven by previous studies, a standardized therapeutic scheme by stage is lacking. In this study, we proposed a new concept of treating Parkinson's disease by stage using combined TCM and WM methods. Then, we designed a multicentre, randomized, double-blind clinical trial using a modified Pingchan formulaformula. The Pingchan formula is a confirmed TCM formula created based on TCM theories and clinical experience. Our team has conducted a battery of trials on the therapeutic effect of Pingchan formula on mild to moderate PD and has confirmed its effect in protecting neurons and reducing the side effects of levodopa. On the basis of past TCM syndrome differentiation studies, we modified the Pingchan formula into the Ziyin Pingchan formula, Jiedu Pingchan formula, and Fuzheng Pingchan formula to treat mild, moderate, and advanced PD patients. To evaluate the therapeutic effect of each formula, we used pertinent scales to verify the specific objectives of the corresponding stage. This therapy combines TCM syndrome differentiation with WM stages, thus establishing a new integrated, long-term management treatment model to delay the progression of the disease in the mild stage and to improve the symptoms, increase the curative effect and reduce the side effects of anti-Parkinson medicine in the moderate and advanced stages.

## Conclusion

This protocol describes the details of a randomized, double-blind, placebo-controlled clinical trial of PD based on TCM syndrome differentiation theories. The results of this study will provide further clinical evidence for the treatment of PD with integrated traditional Chinese and Western medicine and will be used to formulate reliable and manageable individualized treatment for PD patients.

## Data Availability

Not applicable.

## Abbreviations

PD: Parkinson’s disease
TCM: Traditional Chinese medicine
MS: Motor symptoms
NMS: Nonmotor symptoms
QoL: Quality of life
MOA-BI: Monoamine oxidase-B inhibitors
DA: Dopamine agonist
YDLK: Yin deficiency of liver and kidney
DOQB: Deficiency of Qi and blood
PHWS: Phlegm heat and wind stirring
BSWS: Blood stasis and wind stirring
DOYY: Deficiency of Yin and Yang
MPTP: 1-Methyl-4-phenyl-1,2,3,6-tetrahydropyridine
JNK: Jun N-terminal protein kinase
SPIRIT-TCM Extension 2018: Standard protocol items for clinical trials with traditional Chinese medicine 2018
CONSORT: Consolidated standards of reporting trials
IMP: Investigational medicinal product
CRF: Case report form
ICF: Inform consent form
MDS: Movement disorder society
COMT: Catechins-O-Methyl Transferase
PS: Parkinson syndrome
PPS: Parkinsonism-plus syndrome
MoCA: Montreal cognitive assessment scale
Ch. P: The chinese pharmacopoeia
UPDRS: Unified Parkinson’s disease rating scale
PDSS-2: Parkinson’s disease sleep scale-2
ESS: The epworth sleeping scale
RBDSQ: REM sleep behaviour disorder questionnaire
HAMD: Hamilton depression scale
HAMA: Hamilton anxiety scale
SCOPA-AUT: Scales for outcomes in Parkinson’s disease—autonomic
NMSS: Nonmotor symptom scale
PDQ-39: The Parkinson’s disease questionnaire
UA: Uric acid
Hcy: Homocysteine
WCSS: Webster clinical symptom scale
WOQ-9: Wearing-off-9 questionnaire
UDysRS: Unified dyskinesia rating scale
AIMS: Abnormal involuntary movement scale
PDYS-26: Parkinson disease dyskinesia scale
FOGQ: Freezing of gait questionnaire
CBI: Caregivers burden inventory
LED: Levodopa equivalent dose
MMSE: Mini-mental state examination
RBC: Red blood cell count
WBC: White blood cell count
PLT: Platelet count
GOT: Glutamic oxaloacetic transaminase
GPT: Glutamic pyruvic transaminase
Scr: Serum creatinine
EKG: Electrocardiogram
S.D.: Standard deviation
ANOVA: Analysis of variance
GEE: Generalized estimation equation
